# Reduced S-nitrosylation of TGFβ1 elevates its binding affinity toward the receptor and promotes fibrogenic signaling in the breast

**DOI:** 10.1016/j.jbc.2024.108011

**Published:** 2024-11-20

**Authors:** Joshua Letson, Gang Ren, Xunzhen Zheng, Osama Sweef, Yalitza Lopes Corcino, Saori Furuta

**Affiliations:** 1 Department of Cell & Cancer Biology, College of Medicine and Life Sciences, University of Toledo Health Science Campus, Toledo, Ohio, USA; 2 Department of Orthopaedic Surgery, College of Medicine and Life Sciences, University of Toledo Health Science Campus, Toledo, Ohio, USA; 3 Department of Surgery, College of Medicine and Life Sciences, University of Toledo Health Science Campus, Toledo, Ohio, USA; 4 Department of Medicine, MetroHealth Medical Center, Case Comprehensive Cancer Center, Case Western Reserve University School of Medicine, Cleveland, Ohio, USA; 5 Faculty of Science, Department of Zoology, Tanta University, Tanta, Egypt

**Keywords:** TGFβ1, S-nitrosylation, nitric oxide, breast cancer, mammary epithelia

## Abstract

Transforming Growth Factor β (TGFβ) is a pleiotropic cytokine closely linked to tumors. Previously, we pharmacologically inhibited basal nitric oxide (NO) production in healthy mammary glands and found that this induced precancerous progression accompanied by upregulation of TGFβ and desmoplasia. In the present study, we tested whether NO directly S-nitrosylates (forms an NO-adduct at a cysteine residue) TGFβ for inhibition, whereas reduction of NO denitrosylates TGFβ for de-repression. We introduced mutations to 3 C-terminal cysteines of TGFβ1 which were predicted to be S-nitrosylated. We found that these mutations indeed impaired S-nitrosylation of TGFβ1 and shifted the binding affinity towards the receptor from the latent complex. Furthermore, *in silico* structural analyses predicted that these S-nitrosylation-defective mutations strengthen the dimerization of mature protein, whereas S-nitrosylation-mimetic mutations weaken the dimerization. Such differences in dimerization dynamics of TGFβ1 by denitrosylation/S-nitrosylation likely account for the shift of the binding affinities toward the receptor versus latent complex. Our findings, for the first time, unravel a novel mode of TGFβ regulation based on S-nitrosylation or denitrosylation of the protein.

Formation of dense collagenous stroma, termed desmoplasia, is often detected as a hard “lump”and is a hallmark of cancer (1). Desmoplasia, however, may actually happen in non- or pre-cancerous breast tissues, serving as a strong risk factor for breast cancer (2). Desmoplasia is attributed to the emergence of myofibroblasts (MyoFbs) which are highly contractile and secretory stromal cells derived from various types of tissue resident cells. They are present in cancerous as well as precancerous and tumor-adjacent normal tissues and proposed as a key player in tumor initiation (3, 4). MyoFb differentiation is primarily induced by paracrine factors secreted from parenchymal and stromal cells, namely, Transforming Growth Factor β (TGFβ) (5). TGFβ is a family of cytokines that exert pleiotropic functions (6). For its relevance to tumors, however, TGFβ plays complex and dual roles, serving as both a tumor-suppressor and -promoter depending on the stage and tissue type of tumorigenesis (7). TGFβ expression is often elevated in precancerous breast lesions in association with epithelial-to-mesenchymal transition (EMT) (8, 9), suggesting its possible role in the formation of MyoFbs in these lesions (10).

To determine the mechanism of precancerous progression of the breast, we previously inhibited nitric oxide (NO) in healthy mammary glands of wild-type mice (11, 12). NO is a bioactive signaling molecule produced throughout the body, and its aberrant production is linked to different risk factors for breast cancer (13, 14, 15, 16, 17, 18, 19, 20, 21, 22, 23, 24, 25, 26). We found that the basal levels of NO plummeted along with breast cancer progression. Importantly, we saw that pharmacological deprivation of NO in healthy mammary glands induced precancerous progression accompanied by desmoplasia and TGFβ upregulation (11, 12). These findings suggested that NO plays critical roles in suppressing TGFβ activity and that its deficit could lead to precancerous progression of the breast.

In the present study, we explored the mechanism by which TGFβ activity is suppressed by NO. We hypothesized that TGFβ is directly targeted for S-nitrosylation (SNO), a NO-mediated covalent modification of cysteine residues, which would induce certain conformational changes and modulate protein functions (27, 28). To test this possibility, we introduced mutations to 3 C-terminal cysteines of TGFβ1 which were predicted to be S-nitrosylated. We found that mutations at these sites indeed impaired SNO of TGFβ1 and shifted their binding affinity towards the receptor from the latent complex. Further conformational analyses predicted that these SNO-defective mutations would induce stronger dimerization of mature TGFβ1, whereas SNO at these sites would weaken dimerization, possibly accounting for the shifts of the binding affinities. Our results unravel a novel mechanism of regulating TGFβ activity through SNO or denitrosylation that impacts dimerization and protein-protein interactions.

## Results

### NO downregulates TGFβ activity in normal mammary epithelial cells

**Figure 1:**
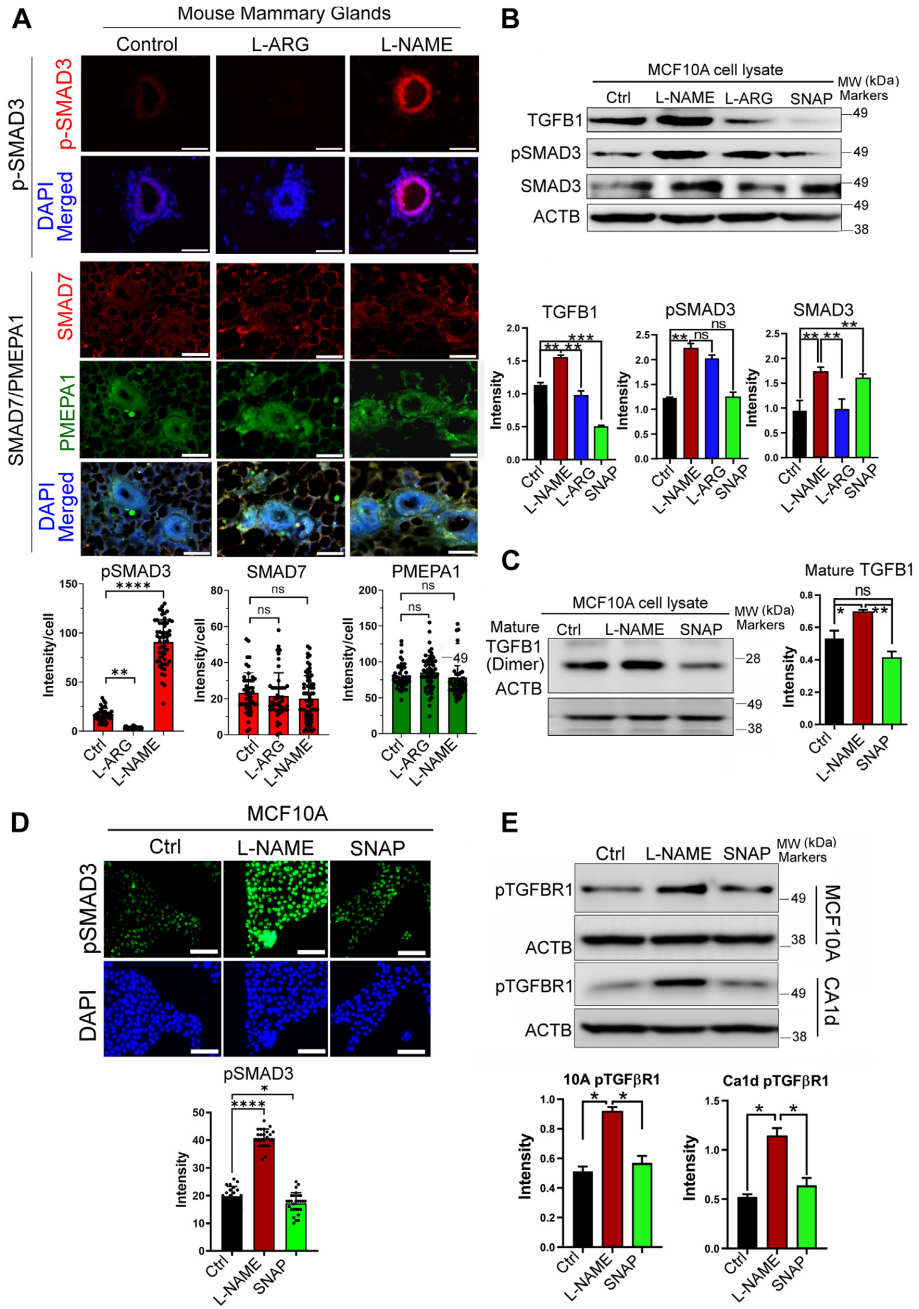
**Inhibition of basal NO production in mammary epithelial cells upregulates TGFβ1 signaling.***A*, (*Top*) Representative images of pSMAD3, SMAD7, or PMEPA1 staining of wild-type mammary glands of drug-treated BALB/c mice. Mice were i.p. treated with PBS (control), L-arginine (20 mg/kg), or L-NAME (20 mg/kg) for 6 weeks (from 4 weeks to 10 weeks old). Nuclei were counterstained with DAPI (*blue*). Scale bars: 50 μm. (*Bottom*) Quantification of the marker intensity per cell in glands. Note a large increase in pSMAD3 signal by L-NAME treatment, in contrast to SMAD7 and PMEPA1 unaffected by treatments. *B*, (*Top*) Western blot showing the protein expression levels of the full-length TGFβ1 and its downstream effector, pSMAD3, in normal human mammary epithelial cells (MCF10 A) following treatment with NOS inhibitor, L-NAME (2.5 mM); NOS agonist, L-arginine (2.5 mM); or NO donor, SNAP (10 μM) for 24 hours. β-actin (ACTB) was used as an internal control. (*Bottom*) Quantification of TGFβ1, pSMAD3 and SMAD3 levels. *C*, (*Left*) Western blot showing the levels of mature TGFβ1 in MCF10 A cells after treatment with L-NAME or SNAP. (*Right*) Quantification of mature TGFβ1 levels. *D*, (*Top*) MCF10 A cells stained with anti-pSMAD3 antibody after treatment with L-NAME or SNAP. (*Bottom*) Quantification of pSMAD3 signals. *E*, (*Top*) WB showing levels of activated TGFβ1 receptor, pTGFBR1, in MCF10 A cells (*top*) and isogenic cancer cells CA1d (*bottom*) after treatment with L-NAME or SNAP. Scale bars: 50 μm. (*Bottom*) Quantification of pTGFBR1 signals in both cell lines. Error bars: ± SEM. ∗, *p* ≤ 0.05; ∗∗, *p* ≤ 0.01; ∗∗∗, *p* ≤ 0.001, and ∗∗∗∗, *p* ≤ 0.0001; *p* > 0.05 was considered significant.

We previously reported that pharmacological inhibition of basal nitric oxide (NO) production with an antagonist, L-NAME, in wild-type mice induced precancerous progression of mammary glands accompanied by highly desmoplastic stroma (11, 12). These glands showed elevated TGFβ activity, as indicated by a strong upregulation of the downstream effector, phospho-SMAD3 (Fig. 1*A*). In contrast, TGFβ/SMAD3 antagonists, SMAD7 and PMEPA1 (29, 30), were not affected by differential NO levels (Fig. 1*A*), indicating the involvement of alternative mechanisms.

To verify this *in vivo* observation, we modulated NO levels *in vitro* using cell lines of MCF10 A human breast cancer progression series, comprising normal MCF10 A and malignant CA1d cells (31). To this end, we applied a NOS inhibitor, L-NAME (2.5 mM), NOS agonist, L-arginine (L-ARG, 2.5 mM), or NO donor SNAP (10 μM) to cultured MCF10 A cells and determined the levels of TGFβ1 and its downstream effector, SMAD3 (phosphorylated and total SMAD3). Consistent to the result of our animal study, we saw a strong negative correlation of NO levels to TGFβ levels and SMAD3 activation. The NO inhibitor, L-NAME, greatly elevated both the full-length and mature form of TGFβ as well as SMAD3 activation (pSMAD3), whereas NO donors, L-arginine (L-ARG) and SNAP, downmodulated all of them (Fig. 1, *B* and *C*). Phosphorylation levels of the receptor, TGFβR1, were concordant with those of SMAD3, attesting to their linear relationship. The same impacts of NO modulators on the receptor activation were observed in both normal MCF10 A and malignant CA1d cells (Fig. 1, *D* and *E*).

**Figure 2:**
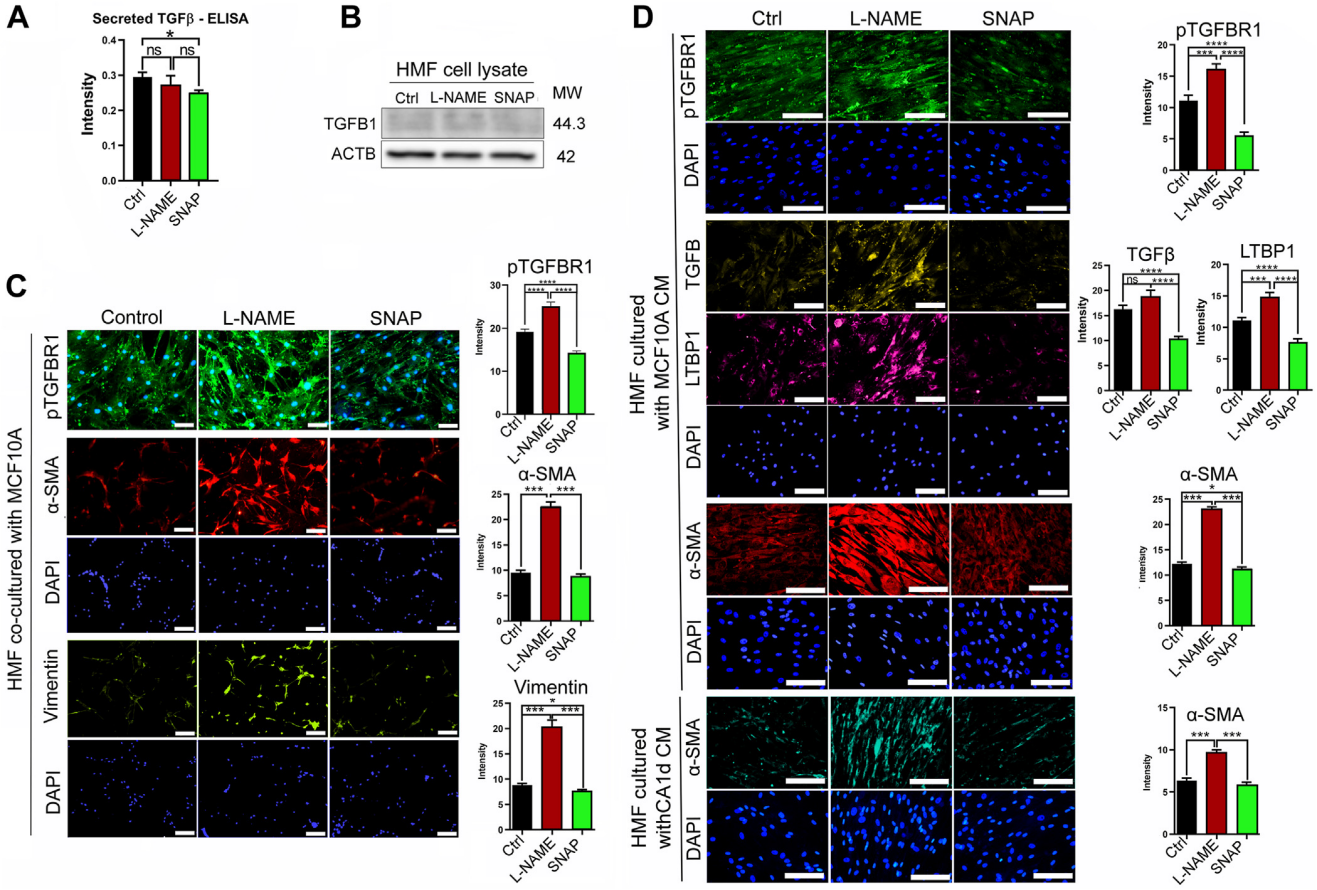
**Paracrine signaling of TGFβ1 signaling is elevated by inhibition of basal NO production in mammary epithelial cells.***A*, ELISA detection of the level of mature TGF β1 secreted from MCF10 A cells after L-NAME or SNAP treatment. *B*, measurement of TGFβ1 levels in human mammary fibroblasts (HMFs) following treatment with L-NAME or SNAP. Note that there is no expression of TGFβ1 in HMFs alone even after treatment with NO modulators. *C*, (*Left*) HMFs co-cultured with MCF10 A cells in the presence of L-NAME or SNAP and stained with antibodies against molecules downstream of TGFβ1 signaling. Scale bars: 50 μm. (*Right*) Quantification of TGFβ1 signaling molecules. *D*, HMFs cultured with the conditioned media (CM) of MCF10 A cells treated with L-NAME or SNAP and stained with antibodies against molecules downstream of TGFβ1 signaling. Scale bars: 50 μm. (*Right*) Quantification of TGFβ1 signaling molecules. Error bars: ± SEM. ∗, *p* ≤ 0.05; ∗∗, *p* ≤ 0.01; ∗∗∗, and *p* ≤ 0.001; *p* > 0.05 was considered significant.

Next, we measured the paracrine signaling of TGFβ in response to modulation of NO levels. ELISA analysis of the conditioned media of drug-treated MCF10 A cells showed that SNAP significantly decreased the levels of secreted total TGFβ, whereas L-NAME did not have any effects (Fig. 2*A*). To test for the differential functionalities of the secreted TGFβ in response to NO modulators, we co-cultured the drug-pretreated MCF10 A cells with human mammary fibroblasts (HMFs, ScienCell, Cat. # 7630). HMFs alone did not express endogenous TGFβ and did not respond to NO modulators (Fig. 2*B*). However, when HMFs were co-cultured with L-NAME-pretreated MCF10 A cells, they significantly elevated phospho-TGFβR1 and downstream fibrotic proteins, α-smooth muscle actin (αSMA), and vimentin. Conversely, HMFs co-cultured with SNAP-pretreated MCF10 A cells significantly downmodulated these proteins (Fig. 2*C*). Alternatively, we cultured HMFs with the conditioned media (CM) from the drug-treated MCF10 A cells. Consistent with the co-culture system, CM of L-NAME-treated MCF10 A cells largely elevated TGFβ signaling molecules in HMFs, whereas CM of SNAP-treated MCF10 A cells downmodulated these proteins (Fig. 2*D*).

### TGFβ1 is S-nitrosylated at specific cysteines to suppress the activity, whereas SNO-defective mutants show increased activity

**Figure 3:**
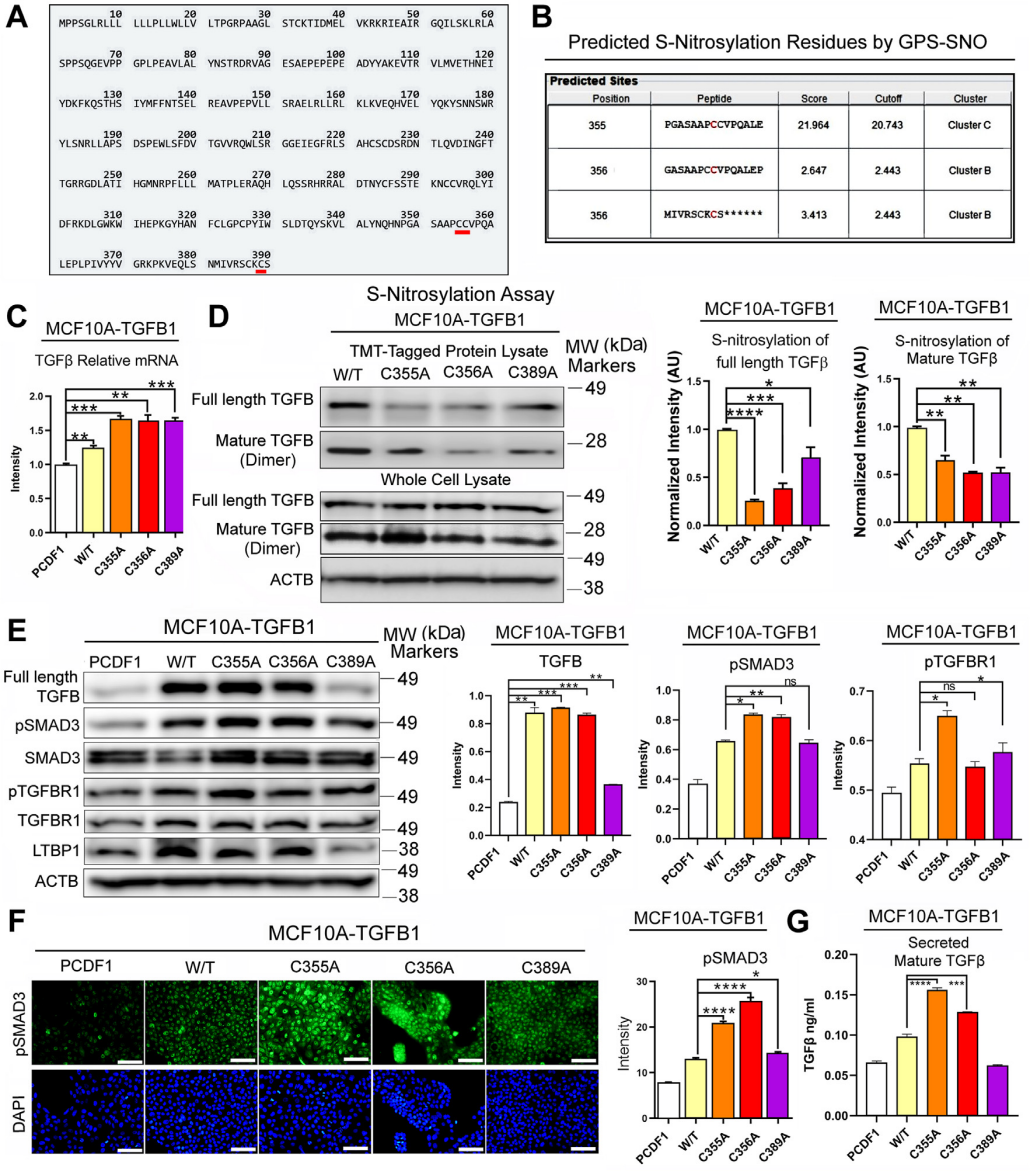
**Impaired S-nitrosylation of TGFβ1 activates its downstream signaling.***A*, full-length TGFβ1 protein sequence showing 3 predicted S-nitrosylation (SNO) sites (highlighted). *B*, probability scores of 3 cysteines predicted to be SNO sites by GSP-SNO software. *C*, TGFβ1 transcripts in MCF10 A cells overexpressing wild-type (WT) or SNO-defective mutants (C355 A, C356 A, or C389 A) of TGFβ1. Note that TGFβ1 transcript levels increased in all the overexpressing cell lines, confirming the expression of the ectopic TGFβ1. *D*, (*Left*) Determination of SNO levels in TGFβ1 overexpressing MCF10 A cell lines (W/T or SNO-defective mutants) by iodoTMT-labeling/pull-down and western blotting against TGFβ1 (full-length and mature) (See the uncut blot for the whole cell lysates in Supplementary Fig. S1*F*.). (*Right*) Quantification of SNO levels of full-length and mature TGFβ1. NOTE that mutant TGFβ1-expressing cell lines showed significantly reduced SNO levels of both full-length and mature TGFβ1. *E*, (*Left*) Western blots showing the levels of TGFβ1 signaling molecules in TGFβ1-overexpressing MCF10 A cell lines (WT or mutants). ACTB was used as an internal control. (*Right*) Quantification of the levels of TGFβ1 signaling molecules in TGFβ1-overexpressing cell lines. *F*, (*Left*) TGFβ1-overexpressing cell lines (WT or mutants) stained with an antibody against pSMAD3. Scale bars: 50 μm. (*Right*) Quantification of pSMAD3 levels in TGFβ1-overexpressing cell lines. *G*, ELISA detection of mature TGFβ1 secreted from MCF10 A W/T or SNO-defective mutants. Error bars: ± SEM. ∗, *p* ≤ 0.05; ∗∗, *p* ≤ 0.01; ∗∗∗, and *p* ≤ 0.001; *p* > 0.05 was considered significant.

To explore the mechanism by which NO suppresses TGFβ activity, we tested our hypothesis that TGFβ is directly targeted for S-nitrosylation (SNO), a NO-mediated covalent modification of cysteines, to suppress its functions. SNO takes place in over 3000 proteins that meet specific physical and biochemical requirements, inducing conformational changes to modulate the protein functions (27, 32). Potential candidate proteins and specific SNO sites could be predicted using software such as GPS-SNO (33). Upon inputting the full-length TGFβ1 protein (390 AAs) sequence into GPS-SNO, the program predicted 3 C-terminal cysteines, C355, C356, and C389, as the potential SNO sites with high probabilities (Fig. 3, *A* and *B*). To validate whether these sites are indeed S-nitrosylated, we introduced point mutations by substituting cysteines with alanines (C355 A, C356 A, and C389 A) and expressed these mutants in comparison to wild-type TGFβ1 and an empty vector (PCDF1) control in MCF10 A cells. Overexpression of these ectopic TGFβ1 proteins were confirmed by RT-PCR (Fig. 3*C*). We also used siRNAs that specifically targeted the endogenous wild-type TGFβ1 to confirm the strong expression of the ectopic proteins (Table S1, Fig. S1, *A–D*). Then, we determined the S-nitrosylation levels of these overexpressing cell lines using the iodoTMT-tagging/pull-down method (34). We indeed saw strong SNO of both full-length and mature TGFβ1 in cells overexpressing the wild-type protein, whereas SNO levels of TGFβ1 significantly declined in cells overexpressing 3 mutant constructs (Fig. 3*D*, Fig. S1*E*). These results strongly indicate that TGFβ1 is S-nitrosylated at these 3 cysteines.

**Figure 4:**
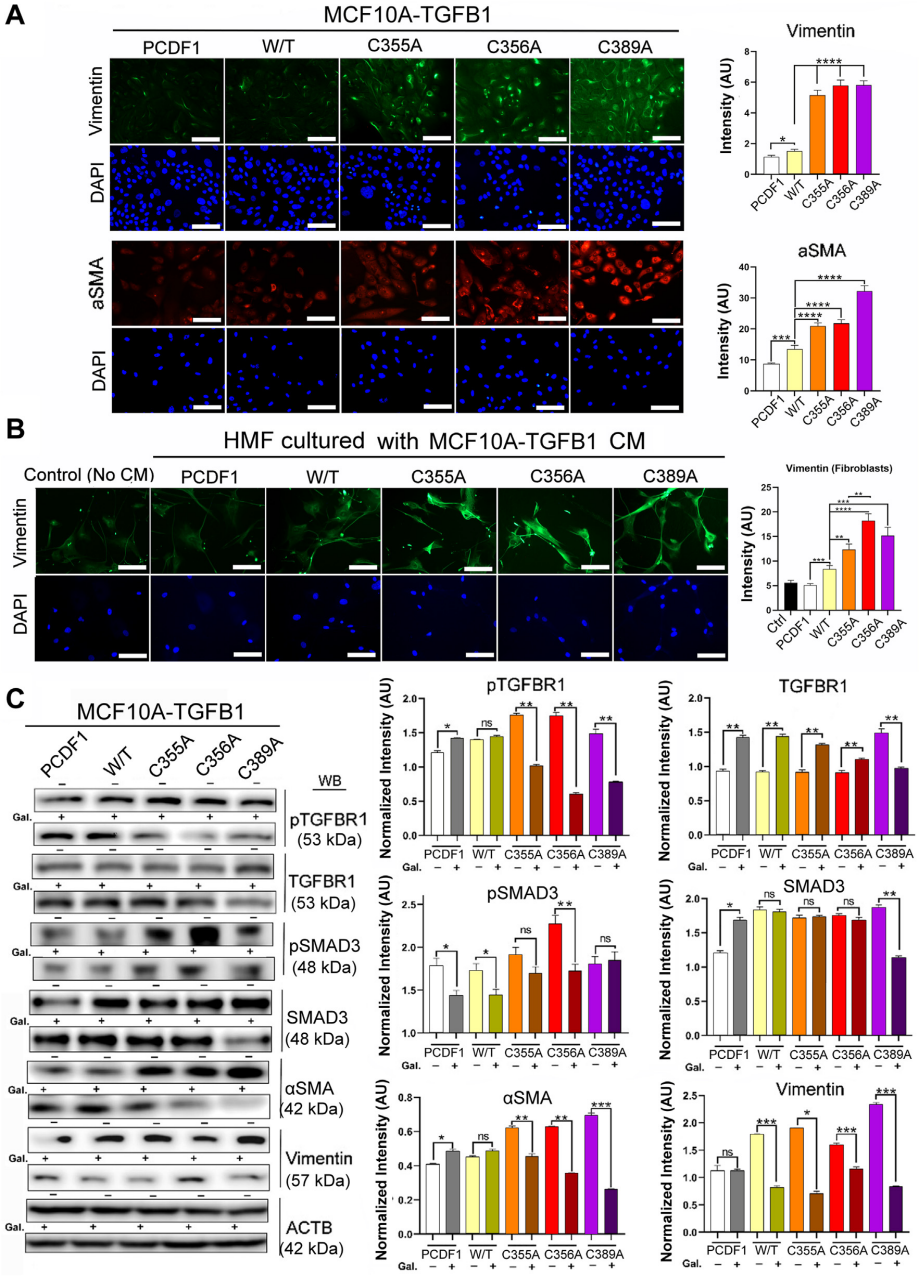
**Impaired S-nitrosylation of TGFβ1 promotes fibrogenic signaling.***A*, (*Left*) TGFβ1-overexpressing MCF10 A cell lines (WT or SNO-defective mutants) stained with antibodies against fibrogenic markers, vimentin and α-smooth muscle actin (αSMA). Scale bars: 50 μm. (*Right*) Quantification of vimentin and αSMA levels in TGFβ1-overexpressing cell lines. Note the significant increases in both fibrogenic markers in SNO-defective mutants. *B*, (*Left*) HMFs cultured with the CM of TGFβ1-overexpressing MCF10 A cell lines (WT or mutants) and stained with an antibody against vimentin. Scale bars: 50 μm. (*Right*) Quantification of vimentin levels in TGFβ1-overexpressing cell lines. *C*, (*Left*) Western blot for the validation of the impacts of TGFβ1-overexpression on fibrogenic signaling using treatment with a TGFβ1 inhibitor galunisertib (Gal, 10 μM, 48 hours). ACTB was used as an internal control (See uncut blots in Supplementary Fig. S1*F*). (*Right*) The levels of fibrogenic molecules normalized against Gal-untreated samples in TGFβ1-overexpressing cell lines with or without Gal treatment. Error bars: ± SEM. ∗, *p* ≤ 0.05; ∗∗, *p* ≤ 0.01; ∗∗∗, and *p* ≤ 0.001; *p* > 0.05 was considered significant.

Next, we sought to investigate the effects of SNO vs. denitrosylation of these sites on downstream signaling. We saw significant increases in TGFβ1 levels and downstream signaling molecules (pSMAD3 and pTGFBR1) in cells overexpressing SNO-defective (denitrosylated) TGFβ1 mutants (Fig. 3, *E* and *F*). Secreted levels of TGFβ were also significantly elevated in SNO-defective cell lines compared to the wild-type construct (Fig. 3*G*). Consistently, fibrotic proteins, αSMA and vimentin, were also elevated in MCF10 A cells overexpressing either of the 3 mutants (Fig. 4*A*) as well as HMFs cultured with CM of these MCF10 A cell lines (Fig. 4*B*). To verify that such induction of fibrotic signaling was indeed caused by TGFβ1, these MCF10 A cell lines were treated with a TGFβ inhibitor, galunisertib (Gal). As expected, Gal treatment compromised the induction of fibrotic signaling by the mutants, further attesting to the critical roles of SNO-defective TGFβ1 in fibrotic activation (Fig. 4*C*, Supplementary Fig. S1*F*).

### SNO of TGFβ1 shifts its binding affinities toward the latent complex, while SNO-defective mutants have affinities towards the receptor due to the difference in dimerization dynamics

**Figure 5:**
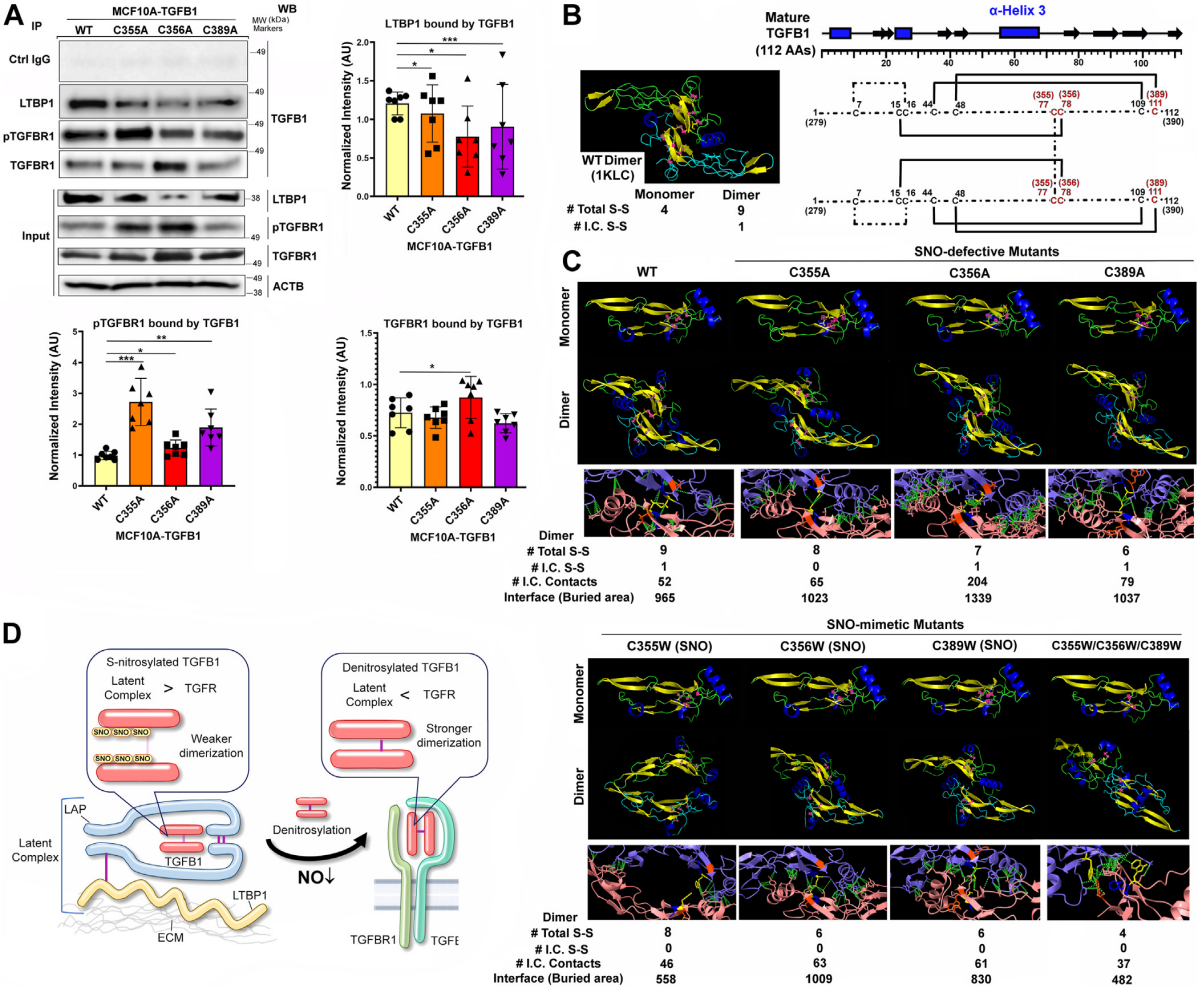
**Impaired S-nitrosylation of TGFβ1 elevates its binding affinities towards the receptor over the latent complex through a change in dimerization dynamics.***A*, (*Left top*) Immuno-precipitation to determine the interaction of W/T or mutant TGFβ1 with the latent complex (LTBP1) vs. receptor (pTGFBR1 and TGFBR1). (*Left middle*) Western blot showing the input levels of LTBP1, pTGFBR1, and TGFBR1. ACTB was used as an internal control. (*Right*) The levels of LTBP1, pTGFBR1 and TGFBR1 normalized against the input levels in TGFβ1-overexpressing cell lines. Error bars: ± SEM. ∗, *p* ≤ 0.05; ∗∗, *p* ≤ 0.01; ∗∗∗, and *p* ≤ 0.001; *p* > 0.05 was considered significant. *B*, (*Left*) The structure of mature TGFβ1 dimer (PDF ID: 1KLC) featuring α-helix 3 (*blue*) and β-strands (*yellow*). (*Right*) Schematic representation of the dimer of mature TGFβ1 (112AAs) featuring intra- and inter-chain disulfide linkages. AA numbers are shown as both the numbers in mature and full-length proteins (parenthesized). SNO sites are shown in *red*. *C*, (First row) Predicted monomeric or dimeric structures of the mature WT and SNO-defective mutants (C355 A, C356 A, or C389 A) of TGFβ1. Chain A: *light green* and Chain B: *cyan*. Secondary structures: α-helix, *blue*; and β-sheet, *yellow*. Disulfide bonds are shown as *redboxes*. (Second row) The interfaces between the 2 monomers. Chain A: *purple*; and Chain B: *pink*. AA 355: *yellow*; AA 356: *blue*; and AA 389: *orange*. Inter-chain contacts: *greendotted lines*. The numbers of the total and inter-chain (I.C.) disulfide bonds (S-S), inter-chain contacts, and interfaces (buried areas, Å) between the 2 monomers are shown. (Third row) Predicted monomeric or dimeric structures of the mature WT and SNO-mimetic mutants (C355 W, C356 W, C389 W, or C355 W/C356 W/C389 W) of TGFβ1. (Fourth row) The interfaces between the 2 monomers. (Please see the whole structures of dimers with the interfacial properties in Supplementary Fig. S2). *D*, working model of the SNO-mediated affinity shift of mature TGFβ1, where SNO shifts the affinity toward the latent complex and denitrosylation shifts it towards the receptor.

To explore the mechanistic basis for SNO-mediated suppression of TGFβ1 activities, we analyzed the interactions of the wild-type vs. SNO-defective TGFβ1 mutants with the latent complex protein, LTBP1, and the receptor, TGFBRI, *via* immuno-coprecipitation. The mature TGFB1 protein forms a homodimer and interacts with the latent protein (LAP) which then binds to LTBP1 to be sequestered in the extracellular matrix (ECM) and binds TGFBRII, which then recruits TGFBRI for receptor dimerization. Thus, the level of TGFB1 binding to TGFBRI, but not TGFBRII, is directly proportional to the strength of the activation signal (35). We saw that SNO-defective mutants showed significantly reduced binding to LTBP1 but increased binding to TGFBRI (both the total and phosphorylated proteins) (Fig. 5*A*). This result suggests that the SNO of TGFβ1 shifts its binding affinity towards the latent complex, whereas denitrosylation (SNO-defective mutations) of TGFβ1 shifts the affinity towards the receptor.

To further investigate the mechanistic basis of this phenomenon, we sought to compare the three-dimensional (3D) conformations of the wild-type vs. SNO-defective TGFβ1 mutants. The published structure of the mature TGFβ1 (112 AAs, PDB ID: 1KLC) showed a symmetrical dimer characterized by centrally clustered β-strands (β-sheets) and parallelly aligned α-helices on the side (Fig. 5*B*, left). Each dimer formed 8 intra-chain and 1 interchain disulfide bonds (Fig. 5*B*, right). The interaction of a TGFβ1 dimer with the receptor (TGFBRII) induces the recruitment of the second receptor (TGFBRI), leading to receptor dimerization necessary for signal transduction (36, 37, 38). Dimerization of TGFβ1 is not required for binding TGFBRII, but necessary for the recruitment of TGFBRI and receptor dimerization for downstream signal transduction (35).

To determine the contributions by the 3 cysteines targeted for SNO, we generated the *in silico* 3D conformation of the 112 aa-mature TGFβ1 protein (Fig. 5*B*, right) using LBL Foldy software which predicts the structure of proteins using AlphaFold 3 modeling platform (39). A monomer with the highest probability score was assembled into a homodimer using HADDOCK 2.4 *ab initio* docking software (40). To attest to the feasibility of this approach, the predicted homodimer of the wild-type protein resembled the published structure (1KLC) forming 8 intra-chain and 1 inter-chain disulfide bond. On the other hand, the predicted homodimers of 3 SNO-defective mutants (C355 A, C356 A, and C389 A) exhibited lower numbers of total disulfide bonds than the wild type. Nevertheless, they all formed higher numbers of inter-chain atomic contacts and larger interfacial surfaces (areas buried by monomeric interactions) than the wild-type (Fig. 5*C*, Fig. S2). Given that interchain contacts and interfacial buried areas are both positively correlated to the binding affinity of interacting proteins (41, 42), these SNO-defective mutants appeared to form dimers with stronger dimerization affinity than the wild type. Complementarily, we analyzed SNO-mimetic mutants where the 3 reactive cysteines were singly or triply changed to tryptophans (C355 W, C356 W, C389 W, and C355 W/C356 W/C389 W) (43, 44). The predicted homodimers of the SNO-mimetic mutants all lacked inter-chain disulfide bonds, and except for the C356 W mutant, they formed dimers that had lower numbers of inter-chain atomic contacts and smaller interfacial areas than the wild-type, indicating their weaker dimerization affinity which was amplified by triple substitution (C355 W/C356 W/C389 W) (Fig. 5*D*, Fig. S2). These shifts in dimerization dynamics through SNO (SNO-mimetic mutations) vs. denitrosylation (SNO-defective mutations) may potentially contribute to the differential binding affinities of TGFβ1 towards the latent complex vs. receptor (Fig. 5*A*). This is in part consistent with the notion that dimerization of TGFβ1 is necessary for the recruitment of TGFBRI to the receptor complex and receptor dimerization for downstream signal transduction (35).

## Discussion

It has been reported that mature TGFβ proteins alter their conformations between “open” and “closed” forms in solution. The closed, compact conformation is formed once 2 α-helices 3 in 2 monomers establish stable interactions and is a favorable condition for receptor binding. However, the predominance of either state depends on a specific TGFβ isoform. For example, TGFβ1 prefers closed over open conformations, whereas TGFβ3 prefers open over closed conformations (36). Interestingly, these biochemical differences in the closed vs. open conformations of TGFβ1 are analogous to the differences we saw in the structures and functionalities of denitrosylated (SNO-defective mutant) vs. S-nitrosylated (SNO-mimetic mutant) TGFβ1. It is thus possible that SNO of mature TGFβ1 helps shift the conformational equilibrium towards the otherwise unfavorable open conformation to weaken the binding affinity towards the receptor. This speculation is in part supported by a report that many cysteines (including those targeted for SNO) of TGFβ1 are critical for the latency and their loss could lead to the constitutive activation of the protein (45).

Our previous and present studies strongly suggest that TGFβ1 is highly S-nitrosylated for latency in normal breast cells. However, as the basal NO production declines possibly due to the increase in oxidative stress, TGFβ1 becomes denitrosylated and derepressed, contributing to the induction of desmoplasia and precancerous progression (11, 12, 27). The roles of TGFβ1 in precancerous progression, however, remain controversial. Classically, TGFβ1 is proposed to exert tumor suppressive roles in early-stage carcinogenesis. In contrast, some more recent studies demonstrated that TGFβ1 acts to stabilize precancerous cells by preventing p53-mediated apoptosis and other stress responses (7, 8, 9, 46, 47, 48). Our studies also strongly indicate that de-repression of TGFβ1, in response to the impairment of basal NO production in the breast, has positive roles in precancerous progression through induction of fibrogenic signaling. Our observation is in line with the well-established roles of TGFβ in EMT-mediated tumorigenesis (8, 9).

Reduced NO bioavailability is associated with different chronic conditions, such as diabetes, cardiovascular disease, and obesity as well as aging. It often leads to the formation of stiff vasculature and desmoplastic ECM, which is directly linked to increased cancer risk (49, 50, 51). Complementarily, moderate to leisure time exercise is shown to elevate basal NO production, ameliorate vascular stiffening, and reduce cancer risks in older people (52, 53, 54). Such NO-associated tissue stiffening is largely attributed to the downmodulation of SNO-mediated inhibition of ECM-remodeling enzymes, including tissue transglutaminases and matrix metalloproteinases (27, 52). In addition, our study reports that a major fibrogenic protein, TGFβ1, is another contributor to tissue stiffening induced by reduction of SNO (11, 12). While in healthy cells a subset of proteins are constitutively S-nitrosylated to remain under control, in disease states, on the other hand, SNO levels could be dysregulated, contributing to disruption of homeostasis (32). The development of novel therapeutics aimed to modulate SNO levels of specific proteins may warrant further investigations.

## Experimental procedures

### Cell lines

Throughout the study, the MCF10 A breast cancer progression series cell lines were utilized consisting of MCF10 A and Ca1d and were obtained from Karmanos Cancer Institute. Primary human mammary fibroblasts (HMFs) were obtained from ScienCell (ScienCell Research Laboratories, Cat. # 7630). 293FT cells were obtained from Thermo Fisher Scientific (Cat. #R70007). Each cell line was validated using short tandem repeat (STR) by the respective vendor before shipment.

### Cell culture

The MCF10 A and Ca1d cell lines were cultured in DMEMF12 (Thermo Fisher Scientific, Waltham, MA, USA, Cat. # 11320033) and supplemented with the following: 5% horse serum (Thermo Fisher Scientific, Cat. # 16050122); 1% penicillin/streptomycin (Thermo Fisher Scientific, Cat. # 15140122); 20 ng/ml EGF (Sigma, Cat. # E−9644); 10 μg/ml insulin (Sigma, Cat. # I-1882); 100 ng/ml cholera toxin (Sigma, St Louis, MO, USA, Cat. # C-8052) and 0.5 μg/ml hydrocortisone (Sigma, Cat. # H-0888). HMF cells cultured in fibroblast basal medium (Sigma, Cat. #115-500) and supplemented with 1% fibroblast growth supplement (Sigma, Cat. #116-GS) and 1% penicillin/streptomycin (Thermo Fisher, Cat. #15140122). All cells were grown in humidified incubator at 37 °C with 5% CO2. 293FT cells were cultured in D-MEM high glucose and supplemented with 10% FBS, 0.1 mM MEM non-essential amino acids, 6 mM L-glutamine, 1 mM MEM sodium pyruvate, 1% penicillin/streptomycin, and 500 μg/ml geneticin.

### Reagents

To inhibit NO production, 2.5 mM of L-NAME (Nω-Nitro-L-arginine methyl ester hydrochloride) was added to cell cultures (Sigma, Cat. #N5751). L-arginine, an NO synthase agonist, was used at the concentration of 2.5 mM and obtained from Sigma (Cat. # A6969). NO donor SNAP (S-Nitroso-N-acetyl-DL penicillamine) was used at the concentration of 10 μM (Sigma, Cat. #N3398). Galunisertib (LY2157299), a TGFβ inhibitor, was used at the concentration of 10 μM and obtained from Cayman Chemical (Cat. # 15312).

### Antibodies

The following antibodies were used for western blotting and ICC/IF: anti-β actin (Sigma, A1978); anti-TGF Beta 1 (Abcam, ab92486); anti-LTBP1 (Abcam, ab78294); anti-phospho-Smad3 (Novus Biologicals, NBP1-77836); anti-phospho-TGFβR1 (Abcam, ab112095); anti-αSMA (Abcam, ab7817); anti-Vimentin (Thermo Fisher, PA5-86264); anti-Histone H3 (Cell Signaling Technologies, 4499S); anti-Smad 3 (Cell Signaling Technologies, 9523S); and anti-TGFβR1 (Abcam, ab31013).

#### Animal studies

All animal experiments conformed to The Guide for the Care and Use of Laboratory Animals (National Research Council, National Academy Press, 2010) and were approved by the Institutional Animal Care and Use Committee of the University of Toledo, Toledo, OH (Protocol No: 108658). Three-week-old female BALB/c (n = 18) mice were obtained from the Jackson Laboratory and housed under a 12 hr light-dark cycle and given regular chow. Starting at the age of 4 weeks old, mice were given intraperitoneal injections of either drug (vehicle: PBS (100 μl), L-arginine (20 mg/kg, 100 μl), or L-NAME (20 mg/kg, 100 μl)) every other day for 6 weeks. The body weight and morbidity of animals were monitored throughout the treatment period. At the end of the treatment period, mice were euthanized, and inguinal mammary glands were harvested and processed for paraffin embedding and sectioning.

#### Immunohistochemistry

To determine the expression of specific markers, paraffin-embedded sections of mouse mammary tissues were analyzed by immunohistochemistry. Briefly, sections were deparaffinized, hydrated, and treated with antigen unmasking solutions (Vector Laboratories, Inc.) or with Tris-EDTA Buffer (10 mM Tris Base, 1 mM EDTA Solution, 0.05% Tween 20, pH 9.0) heated to 95 to 100 °C in a pressure cooker. After being blocked with nonimmune goat serum, sections were processed for immunofluorescence staining as described below.

#### Immunofluorescence staining of tissues and imaging

Immunofluorescence staining/imaging of tissues was performed as described previously (55). Samples were incubated with primary antibody overnight at 4 degrees in a humidified chamber. After intensive washing (3 times, 15 min each) in 0.1% BSA, 0.2% Triton-X 100, 0.05% Tween 20, 0.05% NaN3 in PBS, fluorescence-conjugated secondary antibodies (Molecular Probes) were added for 1 hr at room temperature. Nuclei were stained with 0.5 ng/ml DAPI. After mounted with anti-fade solution, After mounted with anti-fade solution, epi-fluorescence imaging was performed on the Leica Thunder Imaging platform with Leica LAS X Life Science Microscope software.

### Immunofluorescence staining of cultured cells and imaging

Cells were seeded and grown on 12 mm round glass coverslips that were coated in PureCol bovine collagen (Advanced BioMatrix, 5005) according to the manufacturer’s protocol. The coverslips were removed from the dish, added to a humidified chamber, and fixed with 4% paraformaldehyde for 15 minutes. The coverslip was washed in PBS-T 3 times (5 minutes each) and permeabilized for 15 minutes with 0.5% triton-X100 and washed again. Blocking was performed by addition of 3% BSA for 1 hour followed by washing. Primary antibody (1:100-1:200) in 3% BSA/0.1% saponin was added dropwise to the coverslips and incubated overnight at 4 °C. The coverslips were washed 6x before the addition of secondary antibody (1:1000) and left for 2 hours to incubate at room temperature. After washing, nuclei were stained with DAPI (0.5 ng/ml) and incubated for 10 minutes before a final wash. A drop of Fluoromount-G (Thermo Fisher, 00-4958-02) was added to each coverslip and mounted on a glass slide. Fluorescence imaging was performed on an Olympus IX70 microscope with 10x (Numerical Aperture (NA): 0.25), 20x (NA: 0.4), 40x (NA: 0.65) objectives, utilizing CellSens software.

#### Image analysis

Quantification of fluorescence signal in micrographs was performed with ImageJ software (NIH) referring to the owner’s manual (http://imagej.net/docs/guide/146.html). Briefly, a region of interest (ROI) was determined in reference to an image of DAPI-stained nuclei. For quantification of the signal in individual cultured cells, the whole cell was selected as ROI. For quantification of signal in individual organoids in cultures or tissues, each organoid was selected as ROI. For quantification of second harmonics generation (SHG) signal in mammary tissues, the ROI was defined as the periductal ECM/stromal area between the epithelial and adipose layers. For each ROI, the average intensity per pixel was measured, and background intensity was subtracted. For each sample group, at least 50 to 200 measurements were performed. Furthermore, measurement of each sample set was repeated by at least 3 people, and the results were combined for the final data. The mean value was represented as arbitrary units (AU). The statistical significance of the data was further evaluated using GraphPad Prism Version 10 software (see statistics section).

### Western blotting

Cell lysates were prepared by scraping cell culture dish with a plastic policeman after the addition of lysis buffer (25 mM Tris pH 7.4, 150 mM NaCl, 1 mM EDTA, 1% NP-40, and 5% glycerol with the addition of protease and phosphatase inhibitors). The lysate was spun down at 13,000 x g for 15 minutes at 4 °C and the protein lysate was transferred to a new tube. To determine protein concentration, a BCA assay was performed (Thermo Fisher, 23225) according to the manufacturer’s protocol. Reduced samples (by addition of 2-mercaptoethanol) were heated at 95 °C for 5 minutes then loaded onto a 4 to 12% tris-glycine gradient gel (Thermo Fisher, XP04122BOX) and ran at 125 V for 1 to 1.5 hours. Proteins were transferred to a PVDF membrane, and the PVDF membrane was briefly washed in TBS-T and blocked in 5% non-fat dry milk (NFDM) for 1 hour. The membrane was washed 3x for 5 minutes each and the primary antibody was added and incubated overnight at 4 °C. The membrane was probed with HRP-conjugated secondary antibody in 5% NFDM for 1 hour and washed again. The membrane was incubated with Super Signal West Dura substrate (Thermo Fisher, 34076) for 5 minutes and was imaged on a Syngene GBOX (model # Chemi XX6).

### Co-immunoprecipitation

Cells were lysed in RIPA lysis buffer (10 mM Tris-HCl, pH 8.0, 1 mM EDTA, 0.5 mM EGTA, 1% Triton X-100, 0.1% Sodium Deoxycholate, 0.1% SDS, 140 mM NaCl) freshly supplemented with phosphatase inhibitors (1 mM sodium orthovanadate, 30 mM sodium fluoride, 2 mM sodium pyrophosphate, and 10 mM β-glycerophosphate) and protease inhibitor cocktail (0.5 mM Pefa-bloc, 150 nM aprotinin, 1 μM E−64 protease inhibitor, and 1 μM leupeptin; Calbiochem) on ice for 30 minutes. Lysates were centrifuged at 4 °C for 10 minutes at 10,000 g, and the protein concentration was normalized. 0.5 mg protein (1 mg/ml) was precleared with 50 μl protein A/G (1:1) agarose beads (Roche) at 4 °C for 1 hour, then incubated with 2 μg primary antibody at 4 °C overnight and subsequently with 50 μl protein A/G agarose beads for 2 hours at 4 °C. Beads were washed 3 times with 1 ml of the lysis buffer. The immunoprecipitates were boiled in 100 μl SDS sample buffer for 10 minutes, and half the sample volume was analyzed by immunoblotting. 10% of the protein (50 μg) was used for the input analysis.

### Prediction of sites of S-nitrosylation

The amino acid sequence of TGFβ mRNA CDS (NCBI Reference Sequence: NM_000660.7) was analyzed by GPS-SNO 1.0 software to predict which sites of the protein could undergo S-nitrosylation. Cysteine positions 355, 356, and 389 came back as positive targets that could harbor this modification.

### Site-directed mutagenesis of TGFβ

The QuikChange II Site-Directed Mutagenesis Kit (Agilent, 200524) was used according to the manufacturer’s protocol. Primers were designed using Agilent’s QuikChange primer design online software and purchased from Thermo Fisher. The TGF beta 1 (NM_000660) Human Untagged Clone (Origene, SC119746) was used to perform site-directed mutagenesis on residues C355, C356 and C389. Each primer was designed to replace each 3 cysteine residues with an alanine residue. A PCR reaction was performed to create the new sequence with specific mutation sites. The PCR product was visualized on a 1% agarose gel to confirm the proper size. The product was transformed into XL-1 blue supercompotent cells to repair nicks in the DNA strand. The newly transformed XL-1 blue cells were spread onto agar petri plates containing 100 μg/ml ampicillin and incubated upside down in a 37 °C bacterial incubator overnight. Several colonies per plate were picked the next day and added to 5 ml of LB broth containing ampicillin and grown again overnight at 37 °C while rocking at 220 RPM. The broth was collected and centrifuged at 5000*g* to pellet the e-coli cells.

### Ligation into a mammalian expression vector and transformation

A mini-prep (Qiagen, 27104) was conducted on pelleted cells from the transformation completed above following the mini-prep kit protocol. Purified plasmids were analyzed by DNA sequencing and confirmed the amino acid substitution for each site. To this DNA, PCR was performed to create specific restriction sites at the 5′ and 3′ ends of our gene of interest. The restriction sites added were for EcoRI and XbaI. A double restriction digest was performed on the newly synthesized sites to prepare the insert for ligation into the expression plasmid. After the double digest was complete, the products were run on a 1% agarose gel, and the band of interest excised with a razor blade. The DNA was recovered by gel extraction utilizing the QIAquick Gel Extraction Kit (Qiagen, #28704). The DNA concentration of the insert was taken. In order to insert our gene of interest into a mammalian expression vector, we used the PCDF1- MCS2-EF1-Puro expression vector (System Biosciences, CD110B-1). We performed a restriction digest and gel extraction as above to the plasmid. We performed ligation of our insert and expression vector utilizing the T4 DNA ligase (New England Biolabs, M0202S) according to manufacturer protocol. The ligation product was transformed into XL-1 blue supercompotent cells and plated as before. The following morning, we picked several colonies from each clone and grew them overnight in LB broth containing the appropriate antibiotics. We conducted another mini-prep and performed a double digest for EcoRI and XbaI to confirm the size of our insert.

### Transfection, lentivirus production, and transduction

For the generation of pseudoviral particles containing mutant TGFβ1, 293FT cells were cultured and maintained according to the manufacturer's protocol. One day prior to transfection, 293FT cells were seeded to achieve 80% confluency the following day. The transfection was completed following purefection transfection reagent protocol (System Biosciences, LV750 A). Briefly, reduced serum media (Thermo Fisher, #31985-062) was added to a tube along with purification reagent and pPackF1 lentiviral packaging kit plasmids (System Biosciences, # LV100A-1) and either PCF1 (empty vector control), W/T, or point-mutated TGFβ1 plasmids. The mixture was incubated for 15 minutes and added dropwise to plates containing 293FT cells. The media containing pseudoviral particles was collected at 48 and 72 hours and the syringe was filtered to remove any cell debris. The viruses from each time point were pooled and concentrated using a PEG-it virus precipitation solution (System Biosciences, LV810 A) according to the manufacturer’s protocol. For transduction of target cells, MCF10 A cells were seeded to achieve 50 to 70% confluency and the following day the virus particles were added to the dish along with TransDux virus transduction reagent (System Biosciences, LV850 A). The media was changed after 4 days and antibiotic selection was performed using a puromycin concentration of 0.85 μg/ml. The pSIH1-H1-siLuc-copGFP plasmid (System Bioscience, LV601PB) was used as a positive transduction control.

### S-nitrosylation assay

In order to determine the level of s-nitrosylated TGFβ, we used the Pierce S- S-Nitrosylation Western Blot Kit (Thermo Fisher, 90105) following the provided protocol. Briefly, MCF10 A mutant and W/T TGFβ1 expressing cells were grown and harvested in HENS lysis buffer supplied with the kit. Protein concentrations were normalized between samples before beginning the assay. To each sample, methyl methanethiosulfonate (MMTS) was added to block free cysteine thiols. Protein precipitation was performed with ice-cold acetone to remove excess MMTS. The samples were centrifuged, acetone was removed, and the pellet was resuspended in HENS buffer. Sodium ascorbate and the labeling tag (iodo-TMT) were added and the samples were incubated. Protein precipitation was again performed and the pellet was resuspended in the HENS buffer. In order to separate labeled from non-labeled proteins, samples were incubated with immobilized Anti-TMT Resin (Thermo Fisher, 90076). The eluted samples were run on SDS-PAGE for analysis.

### TGFβ measurement

To determine the amount of secreted TGFβ from the MCF10 A cell line, we used the Human TGF beta 1 ELISA Kit (Abcam, ab108912). On Day 1, cells were seeded in normal media. On Day 2, roughly 24 hours later, the serum media was aspirated from the culture plates and serum-free media was added. The cells were left to grow until they reached approximately 90% confluency. The media was collected with a 10 ml syringe and passed through a 0.45-μM filter to remove any cell debris. The media was concentrated by adding the sample to a 3kd cutoff protein concentrator and centrifuged. Once the media had decreased to roughly 1/10 its original volume, the fraction containing the concentrated protein was collected. Protein concentration was taken and samples were normalized against each other. ELISA was performed according to the manufacturer’s protocol.

### Co-culture and conditioned media experiments

Briefly, fibroblast cells were added on top of purecol coated coverslips in the bottom of a 12-well dish with a 1:1 ratio of MCF10 A cell culture media to fibroblast media. We placed the insert on top and added MCF10 A cells along with more growth media. To treat the cells, drugs were added to the top insert. Coverslips were removed after treatment for 48 hours and cells were stained following the staining protocol. For conditioned media experiments, we drug-treated MCF10 A cells and let them grow for an additional 24 hours. After 24 hours of incubation, media was collected from MCF10 A culture plates with a syringe and filtered using a 0.45 μM filter to remove cell debris. At the same time, we aspirated media from fibroblast culture plates and added the drug-treated MCF10 A conditioned media to the plates. We repeated this once more for a total of 2 treatments. After conditioned media treatments on fibroblasts were complete, we harvested the coverslips and stained the cells following our protocol.

### RT-PCR

To determine mRNA levels of TGFβ in our mutant cell lines, we cultured cells and collected RNA utilizing an RNeasy Kit (Qiagen #74004) and created cDNA using SuperScript IV Reverse Transcriptase (ThermoFisher, #18090010) and followed the manufacturer’s protocol. We then performed PCR on cDNA and ran the products on a 1% agarose gel to visualize intensity of the products. (For primers see Table S1).

### Prediction of 3D monomeric and dimeric structures of TGFβ1

C-terminal 112 AA sequence of mature TGFβ1 (wild-type and different mutants) was input into LBL Foldy software (https://foldy.lbl.gov/) (39) which predicts the structure of proteins using AlphaFold 3 modeling platform. The resultant structures (PDB format) with the highest probability scores were further assembled into homodimers using HADDOCK 2.4 *ab initio* docking software, which was also freely available online (https://rascar.science.uu.nl/haddock2.4/) (40). The predicted monomeric or dimeric structures were viewed and analyzed by UCSF ChimeraX (56) and Pymol (57) to determine the number of interchain and intrachain disulfide bonds, the number of interchain contacts, and buried areas (Å^2^, interface) between interacting monomers (https://www.rbvi.ucsf.edu/chimerax/docs/user/selectcontacts.html).

### Statistical methods

All the experiments were performed in replicates (n ≥ 3) and, unless otherwise indicated, two-tailed t-tests were performed to obtain the statistical significance of the mean difference. The statistical significance of the mean difference was tested by parametric tests using GraphPad Prism version 9. *P* value of 0.05 or lower was considered significant and the average value is presented as the arithmetic mean ± SEM.

## Data availability

All study data are included in the article and/or SI Appendix.

## Supporting information

This article contains supporting information.

## Conflict of interest

The authors declare that they have no conflicts of interest with the contents of this article.
