# Supplementary material for: Reduced S-nitrosylation of TGFβ1 elevates its binding affinity toward the receptor and promotes fibrogenic signaling in the breast
Source: J Biol Chem. 2024 Nov 20;300(12):108011. doi: 10.1016/j.jbc.2024.108011 (PMC11699740; doi:10.1016/j.jbc.2024.108011)
Supplement: Supplementary Table and Figures [file mmc1.pdf]

Supplementary Data

Reduced S-nitrosylation of TGFβ1 elevates its binding affinity towards the receptor and promotes fibrogenic signaling in the breast

Joshua Letson, Yalitza Lopes Corcino, Osama Sweef, and Saori Furuta

Table of contents

Supplementary Table S1.. 2

Supplementary Figure S1... 3

Supplementary Figure S2……………………………………………………………………...4

Supplementary Table

**Point Mutation Primers**	**5' to 3' Sequence**
**C355A Point Mutation- Sense**	GCGGCACGCAGGCCGGCGCCGCCG
**C355A Point Mutation- Antisense**	CGGCGGCGCCGGCCTGCGTGCCGC
**C356A Point Mutation- Sense**	CCTGCGGCACGGCGCACGGCGCCG
**C356A Point Mutation- Antisense**	CGGCGCCGTGCGCCGTGCCGCAGG
**C389A Point Mutation- Sense**	GGGACCTCAGCTGGCCTTGCAGGAGCGC
**C389A Point Mutation- Antisense**	GCGCTCCTGCAAGGCCAGCTGAGGTCCC
**TGFβ siRNA**	5' to 3' Sequence
**siRNA 1 Sense**	UCCGUGGGAUACUGAGACAUU
**siRNA 1 Antisense**	UGUCUCAGUAUCCCACGGAUU
**siRNA 2 Sense**	GGGCUGUAUUUAAGGACACUU
**siRNA 2 Antisense**	GUGUCCUUAAAUACAGCCCUU
**siRNA 3 Sense**	CAUUAAAGAUGGAGAGAGGUU
**siRNA 3 Antisense**	CCUCUCUCCAUCUUUAAUGUU
**RT-PCR Primers**	5' to 3' Sequence
**TGFβ Sense**	CTATTGCTTCAGCTCCACGG
**TGFβ Antisense**	CAGTAGTGTTCCCCACTGGTC
**18S Sense**	CGCGGTCCTATTCCATTATTC
**18S Antisense**	CCCGAAGCGTTTACTTTGAAA

Table S1: Primers and siRNAs used in experiments (See Materials and Methods).

Supplementary Figure

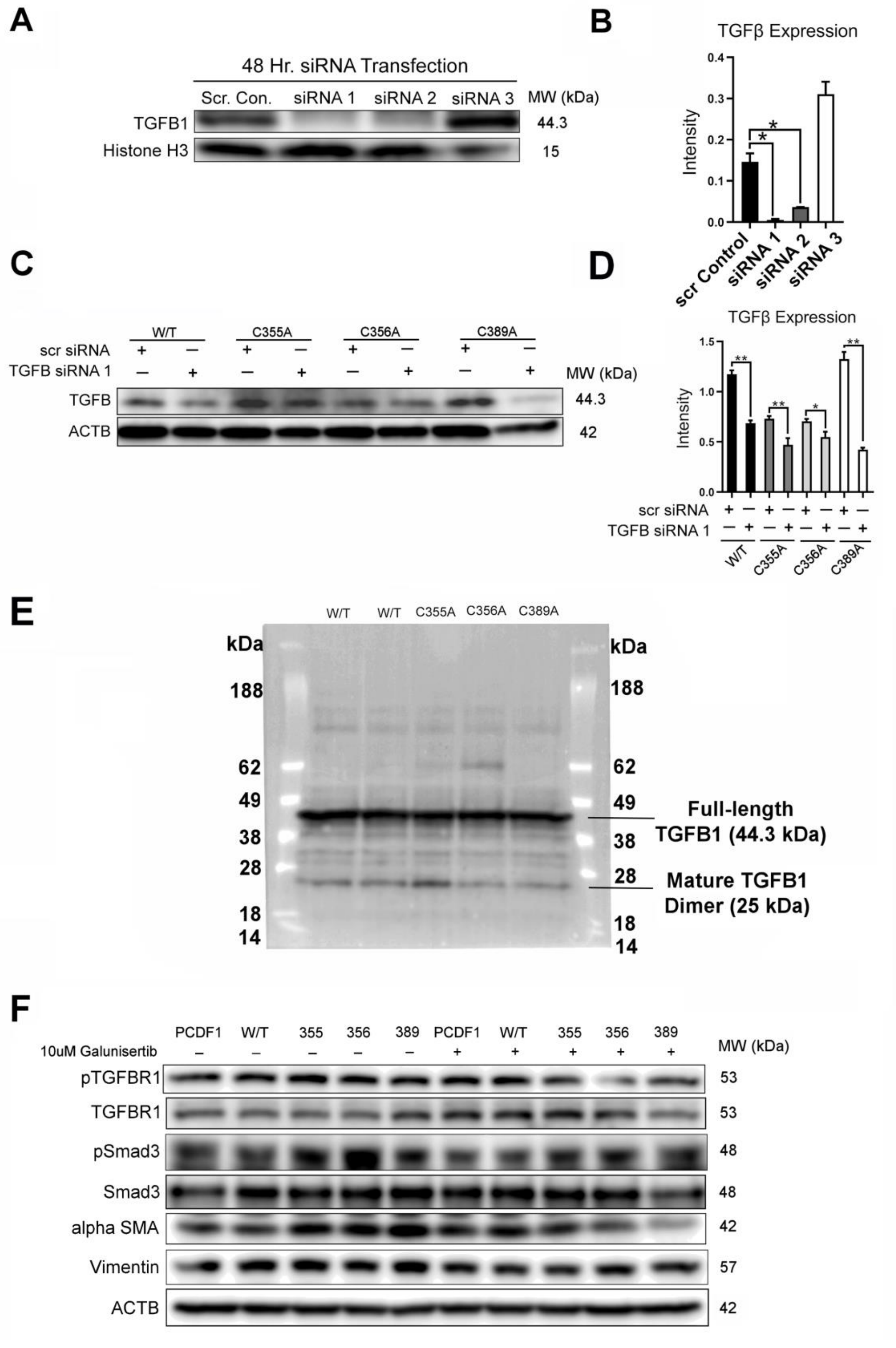

Figure S1: Treatment with siRNAs specifically removing the endogenous TGFβ1 (binding to either 3' or 5' UTR region of mRNAs) shows the levels of ectopic proteins.

A) Western blot for the expression of TGFβ1 in MCF10A cells after treatment with three different siRNAs that target 3' or 5' UTR of the TGFβ1 transcripts. ACTB was used as an internal loading control. **B**) The intensities of TGFβ1 normalized against ACTB. Note that siRNA1 shows the strongest inhibitory effect. **C**) MCF10A cell lines expressing wild-type or SNO-defective mutants (C355A, C356A, or C389A) were treated with either scramble (scr) siRNA or siRNA1 (the most potent siRNA), and TGFβ1 levels were determined by Western blot. ACTB was used as an internal loading control. **D**) The intensities of TGFβ1 normalized against ACTB. Note that even after siRNA treatment, the majority of TGFβ1 proteins remained intact, indicating that they were ectopically expressed proteins. Error bars: ± SEM. *, and $p \leq 0.05$. $p > 0.05$ was considered significant. **E**) The raw data for the western blot in **Fig. 3D** showing the full length and mature TGFβ1 along with molecular markers. **F**) Uncut blots of **Fig. 4C** showing both untreated and galunisertib (Gal)-treated samples.

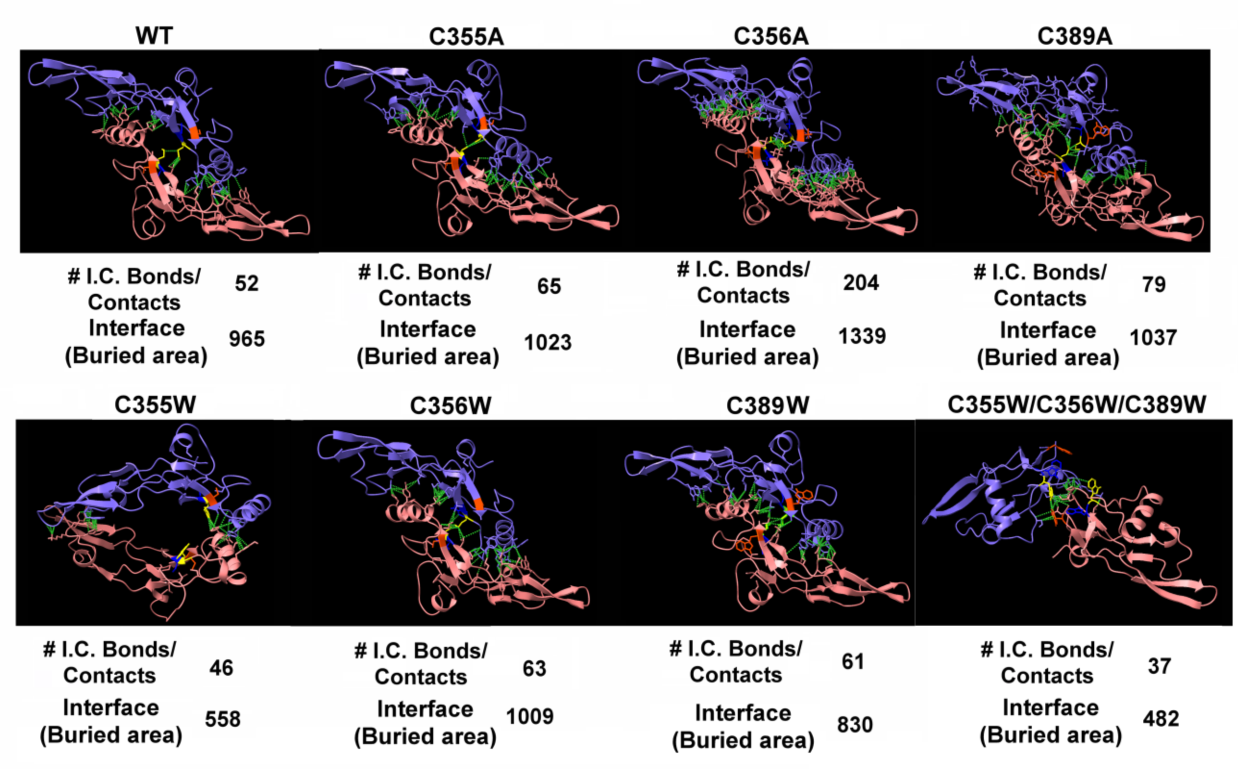

Supplementary Figure S2. Predicted 3D structures of wild-type and mutant forms of mature TGFβ1 dimers.

(Top row) Predicted dimeric structures of the mature WT and SNO-defective mutants (C355A, C356A, or C389A) of TGFβ1. Chain A: purple; and Chain B: pink. AA 355: yellow; AA 356: blue; and AA 389: orange. Inter-chain contacts: green dotted lines. The numbers of the total, inter-chain (I.C.) inter-chain contacts and interfaces (buried areas, Å) between the two monomers are shown. (Bottom row) Predicted dimeric structures of the mature WT and SNO-mimetic mutants (C355W, C356W, C389W, or C355W/C356W/C389W) of TGFβ1.
